# Physical activity is associated with better global cognition and frontal function in overweight/obese older adults with metabolic syndrome

**DOI:** 10.1186/s11556-019-0229-y

**Published:** 2019-12-06

**Authors:** Nina Coll-Padrós, María León, Natalia Valech, Emilio Ros, Josep Vidal, Ramon Estruch, Montserrat Fitó, Jordi Salas-Salvadó, Dolores Corella, José Luis Molinuevo, Lorena Rami

**Affiliations:** 1 Alzheimer’s Disease and Other Cognitive Disorders Unit, Neurology Service, Institut d’Investigacions Biomèdiques August Pi Sunyer (IDIBAPS), Hospital Clínic, Carrer Villarroel 170, 08036 Barcelona, Spain; 2 Lipid Clinic, Department of Endocrinology and Nutrition, IDIBAPS, Hospital Clinic, Barcelona, Spain; 3 CIBER Fisiopatología de la Obesidad y Nutrición (CIBEROBN), Instituto de Salud Carlos III (ISCIII), Madrid, Spain; 4 Department of Endocrinology and Nutrition, IDIBAPS, Hospital Clínic, Barcelona, Spain; 5 CIBER Diabetes y Enfermedades Metabólicas Asociadas (CIBERDEM), ISCIII, Madrid, Spain; 6 Department of Internal Medicine, IDIBAPS, Hospital Clínic, Barcelona, Spain; 7 Cardiovascular Risk and Nutrition Research Group, IMIM-Institut de Recerca del Hospital del Mar, Barcelona, Spain; 8 Human Nutrition Unit, University Hospital of Sant Joan de Reus, Department of Biochemistry and Biotechnology, Pere Virgili Institute for Health Research, Rovira i Virgili University, Reus, Spain; 9 Genetic and Molecular Epidemiology Unit, School of Medicine, University of Valencia, Valencia, Spain; 10 Barcelona βeta Brain Research Center, Pasqual Maragall Foundation, Barcelona, Spain

**Keywords:** Cognitive function, Physical activity, Aging, Metabolic syndrome, Obesity, PREDIMED-PLUS study

## Abstract

**Background:**

There is epidemiological evidence of an association between the metabolic syndrome (MetS), a cluster of cardiovascular risk factors related to central adiposity and insulin resistance, and cognitive impairment and dementia. On the other hand, there is evidence for a beneficial effect of physical activity on cognitive outcomes in older adult populations. In a cross-sectional study, we evaluated the relationship between aerobic physical activity and cognition in a cohort of overweight/obese older adults with MetS at risk for dementia. Cognitive function was assessed in a subsample of 82 subjects (men 55–75 y; women 60–75 y), with MetS and a BMI ≥27 to < 40 kg/m^2^ enrolled in the PREDIMED-PLUS study, a trial of diet and exercise in individuals with MetS with outcomes of cardiovascular prevention. Domain Z scores were calculated for the different cognitive domains. Aerobic physical activity was determined with the Rapid Assessment of Physical Activity questionnaire.

**Results:**

Adjusted covariance analyses revealed that, compared to sedentary participants, those physically active obtained higher scores in mean global cognitive scores, with mean adjusted difference 0.254 (95% CI 0.032 to 0.477, *p* = 0.026) and frontal composites, with mean adjusted difference 0.375 (95% CI 0.110 to 0.639, *p* = 0.006).

**Conclusions:**

Our findings indicate that aerobic physical activity is associated with better global cognition and frontal function in overweight/obese older individuals with MetS, suggesting that physical activity could be a therapeutic strategy to reduce the risk of developing cognitive impairment or dementia in this population.

## Background

In the last decades, the number of persons living with chronic age-related conditions, such as dementia, diabetes or hypertension, has enormously grown as a consequence of the increased life expectancy and the global aging of the population, and these population changes are already having a considerable impact on social and public health systems [1].

The metabolic syndrome (MetS) refers to a cluster of cardiometabolic factors, including excess abdominal fat, high blood pressure, high blood sugar, high triglyceride levels and low HDL-cholesterol. Sedentary lifestyle habits and increasing obesity rates explain an epidemic growth of MetS prevalence worldwide. The available evidence indicates that presently close to 30% of the adult population worldwide harbours the MetS [2]. MetS prevalence is strongly related to age: by age 60, 46.7% of the U.S. population was affected [3]. The MetS has long been known to be associated with an increased risk of cardiovascular disease and type-2 diabetes; but in recent years evidence has accumulated that individuals with MetS are also at high risk for developing neurological conditions such as cognitive impairment and dementia [4–6].

Despite that many studies have reported an association between MetS and poor cognitive function [7–13], controversy still exists. For example, a recent systematic review and meta-analysis of 13 longitudinal population-based studies found a weak association of MetS with cognitive decline, but this relationship was not observed in the older age group (> 70 years) when an age-stratified analysis was performed [14], concurring with other reports suggesting that the association between MetS and poor cognitive function does not hold in older populations [9, 15, 16]. In line with these findings, some studies indicate that the risk of developing dementia is stronger when exposure to risk factors such as obesity, diabetes or hypertension occurs in mid-life rather than in late life [17–19].

Similarly, the evidence for the effect of physical activity on cognition in older adults is still a topic under debate. While three meta-analyses of prospective studies found a positive effect of physical activity on cognition [20–22], another systematic review reported no cognitive benefit [23], and a recent large prospective cohort study with a 28-year follow-up suggested that physical activity in midlife is not associated with a reduced risk of dementia and that previous findings of a lower risk of dementia in physically active individuals might be attributable to reverse causation [24].

In recent years, increasing evidence shows that, compared to healthier individuals, those at an elevated risk of dementia are the ones who obtain greater health benefits from lifestyle interventions, therefore, preventive lifestyle interventions such as physical activity might result in better outcomes (e.g. significant cognitive improvement) when targeted to individuals who are at an increased risk of developing dementia (e.g. overweight/obese older adults with MetS) [25].

In this sense, it is worth noting that most epidemiological studies examining the effect of physical activity on cognitive outcomes have been carried out in populations of healthy older adults. Caution must be taken before generalizing study findings, as individuals with different risk profiles might obtain different results from similar lifestyle interventions.

The impact of physical activity on cognition in overweight/obese older individuals with MetS has not been studied. In fact, there are only a handful of studies on physical activity and cognition conducted in similar populations, e.g. patients with type-2 diabetes, insulin resistance or impaired glucose tolerance, but the evidence available so far does not seem to be robust enough to conclude that physical activity or exercise interventions contribute to a better cognitive performance in these patient groups [26].

In the present study we assessed the relationship between physical activity and cognition in a cohort of overweight/obese older adults with MetS at risk for dementia. We hypothesized that individuals who regularly engage in physical activity would perform better in a cognitive battery compared to individuals with no or very low physical activity.

## Methods

### Study subjects

This study is a cross-sectional analysis of baseline data acquired in a subsample of individuals enrolled in the PREDIMED-PLUS trial, a multicenter, randomized, parallel-group, clinical trial using a low-energy Mediterranean diet plus increased physical activity for the primary prevention of cardiovascular diseases (for more information, please refer to www.predimedplus.com). The local Ethics Committee approved the study and all participants provided written informed consent prior to enrolment.

Eligible candidates were community-dwelling men (aged 55 to 75 years) and women (aged 60 to 75 years) with overweight or obesity [body mass index (BMI) ≥27 to < 40 kg/m2] meeting at least three criteria for the MetS according to the updated harmonized criteria of the International Diabetes Federation and the American Heart Association and National Heart, Lung and Blood Institute [27]. Exclusion criteria were illiteracy, inability to provide written informed consent, history of any cardiovascular disease, active cancer or malignancy within the last 5 years, inability to follow the prescribed diet or attend study visits, participation in other weight loss programs in the 6 months before screening, history of surgery for weight loss, bowel resection or inflammatory bowel disease, obesity of endocrine origin, allergy to any food components of the Mediterranean diet, HIV, cirrhosis, alcohol abuse or addiction, serious psychiatric disorders, severe co-morbid condition with less than 24 months’ life expectancy, major organ transplantation, concurrent treatment with immunosuppressive drugs or systemic corticosteroids, or weight loss medication.

The sample for the present cognition study was identified through convenience sampling methods. Between February 2015 and December 2016 PREDIMED-Plus participants recruited in the 2 nodes based at Hospital Clinic were assessed for eligibility. Specific exclusion criteria for the cognition study were: Mini Mental State Examination (MMSE) < 26 [28]; obtaining abnormal scores (< 1.5 SD below the normative mean) on at least two of the neuropsychological tests performed; insufficient command of the Spanish language, having a serious/unstable neurological disease; taking psychoactive medication (benzodiazepines, opioids …); history of significant head trauma or brain surgery; history of major depression or prior chemotherapy, and suffering claustrophobia or having body implants incompatible with magnetic resonance imaging (MRI).

### Physical activity

Physical activity was estimated with the Rapid Assessment of Physical Activity (RAPA) questionnaire [29], an easily administered tool for assessing level and intensity of physical activity among adults older than 50 years. The questionnaire has two sections: RAPA1 contains seven items measuring aerobic physical activities and RAPA2 contains two items measuring strength and flexibility. For the purpose of this study we focused solely on the RAPA1 (aerobic physical activity), with scores ranging from 1 (“I never do physical activity”) to 7 (“I do 75 minutes or more a week of vigorous physical activities”).

### APOE genotype

Genomic DNA was extracted from buffy coat. We analyzed the common *APOE* polymorphism for all study participants on a 7900HT Sequence Detection System (Applied Biosystems¸ ABI; Foster City, CA, USA) by using fluorescent allelic discrimination TaqMan assays. Two APOE SNPs, rs429358 (Cys112Arg) and rs7412 (Arg158Cys), were selected for genotyping according to the NCBI SNP database (http://www.ncbi.nlm.nih.gov/SNP). The two APOE SNPs (call rate 99%) were further combined to derive the six APOE genotypes (ɛ2/ɛ2, ɛ2/ɛ3, ɛ3/ɛ3, ɛ3/ɛ4, ɛ4/ɛ4 and ɛ2/ɛ4). ɛ2/ɛ4, ɛ3/ɛ4 and ɛ4/ɛ4 were grouped as carriers of the ɛ4 allele (risk genotype) and so-called APOE4 carriers. Genotype frequencies did not deviate from Hardy-Weinberg equilibrium expectations.

### Neuropsychological assessment

Participants underwent a complete neuropsychological assessment comprising all cognitive domains, which included the following tests: Rey Auditory Verbal Learning Test (RAVLT) [30], Rey-Osterrieth complex figure (ROCF) [31] copy and immediate recall, Semantic verbal fluency, Fragmented letters and Number location subtests of the Visual Object and Space Perception Battery [32], Trail Making Test (TMT) parts A & B [33], Symbol Digit Modalities Test (SDMT) [34], Stroop test [35] and WAIS IV digit span [36]. Depression symptomatology was collected with the Hospital Anxiety And Depression Scale (HADS) [37].

## Statistical analyses

The characteristics of the two groups were compared by using Student’s t-test, Mann-Whitney or chi-square tests, as appropriate. We created domain composite cognitive measures by converting the individual test results to Z scores and computing the average Z scores within cognitive domains. The memory composite included the mean standardized individual scores of the RAVLT total learning and recall plus the recall of the ROCF. The frontal function composite included tests measuring attention, cognitive flexibility and working memory, and was built by averaging standardized scores of the TMT, SDMT, Stroop and WAIS IV digit span. The perception composite included the mean standardized individual scores of the VOSP fragmented letter and number location subtests. Language domain score included the standardized individual scores of the semantic fluency test and the praxis domain score included the standardized individual scores of the ROCF copy. Finally, a global cognition composite score was generated by computing the mean standardized scores of all measures.

Shapiro-Wilk normality test results indicated that Language, Praxis and Perception domain scores followed a non-normal distribution. For this reason, Language domain score was transformed into a logarithmic variable, and we confirmed that this transformation normalized data distribution. With regards to the Praxis and Perception domain scores, we determined it was not possible to make any valid conclusions with the data due to very limited variability (ceiling effects) and therefore those two scores could not be analysed.

Two groups were defined according to our sample RAPA1 aerobic physical activity median score: no or low physical activity (≤3) vs. regular weekly physical activity of moderate to vigorous intensity (> 3). We used covariance analyses to compare the global, memory, frontal function and language cognitive domain scores between the two groups: no or low physical activity vs. regular physical activity. Analyses were adjusted for Framingham risk score (encompassing sex, age, total cholesterol, HDL-cholesterol, systolic blood pressure and smoking status) [38] plus hypertriglyceridemia [defined as triglycerides> = 150 mg/dL or fibrate treatment], diabetes [defined by clinical history and/or use of antidiabetic medication], BMI, education years, HADS depression score and presence of the APOE4 genotype. Abdominal obesity [waist circumference > =102 cm in men; > = 88 cm in women] was not included as adjustment variable because 95.1% of the study sample had this MetS component. Statistical significance was set at the <.05 level for all analyses, conducted with IBM SPSS software v. 22.

## Results

**Fig. 1:**
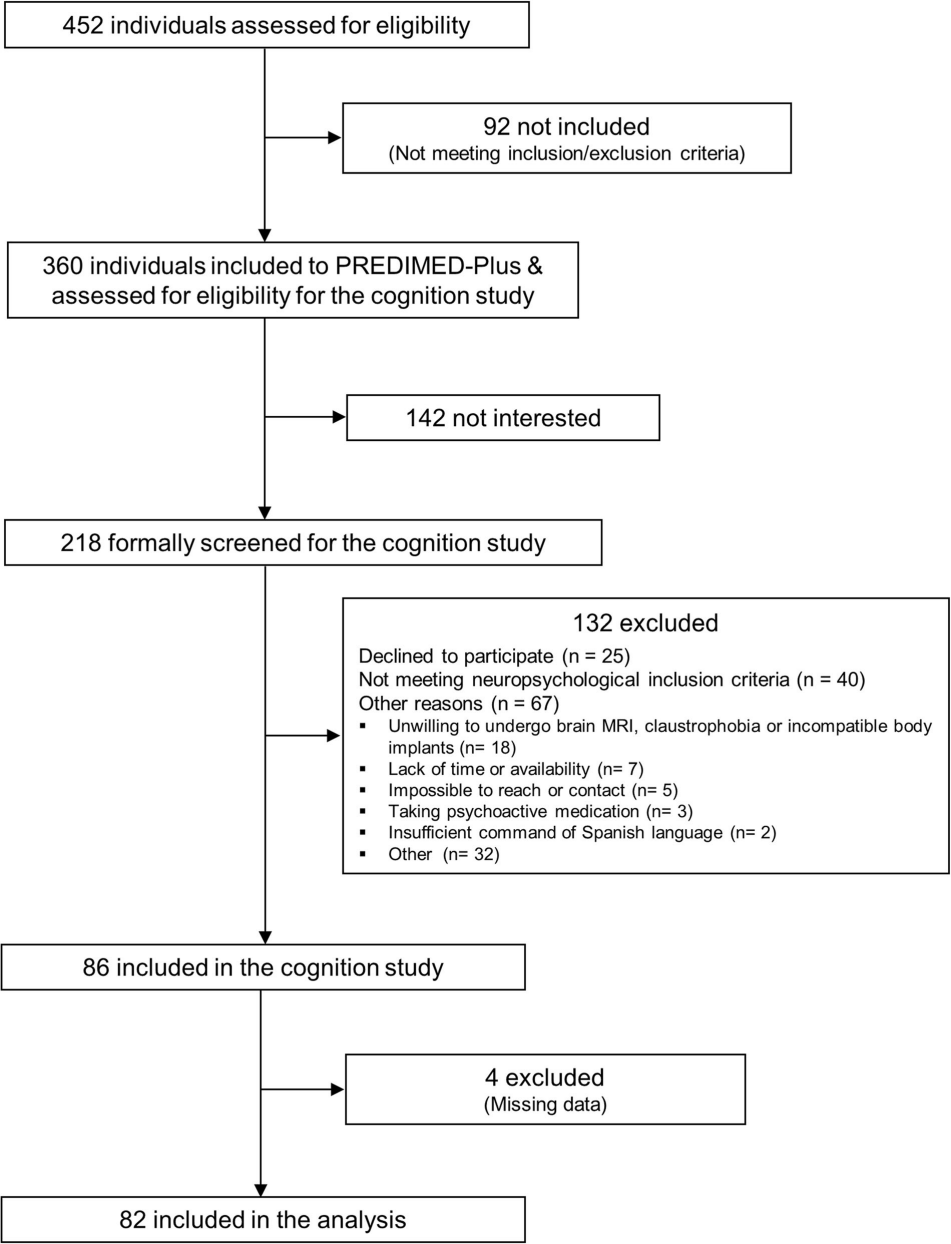
Flow of participants

For details of the flow of participants see Fig. 1. The study sample consisted of 82 individuals (51.2% women), mean age 66.8 ± 4.7 years and mean education 11.7 ± 4.2 years. The mean MMSE score was 29.11 ± 1.08 and mean BMI 32.0 ± 3.07 kg/m2. Regarding MetS components, 95.1% had abdominal obesity, 87.8% had hypertension, 56.1% had hypertriglyceridemia, 50.0% had low HDL-cholesterol, and 65.9% had hyperglycemia or diabetes. In addition, 18.3% had a diagnosis of type-2 diabetes, 46.4% were either current or past smokers and 13.4% were APOE4 carriers. Participants were split into 59 with no or low physical activity and 23 with regular physical activity. The two groups were comparable in most characteristics, except for a higher proportion of women and of individuals with high blood pressure in the low physical activity group (Table 1).

**Table 1:** Subjects’ characteristics by physical activity level

	No or low physical activity *n* = 59	Regular physical activity *n* = 23	*P* value^*^
Sex (women)	59.3%	30.4%	0.028
Age, years (mean, SD)	66.4 (4.68)	67.8 (4.63)	0.231
Education, years (mean, SD)	11.8 (4.37)	11.7 (4.00)	0.962
MMSE (mean, SD)	29.1 (0.98)	29.1 (1.31)	0.661
BMI, kg/m2 (mean, SD)	32.3 (3.12)	31.2 (2.84)	0.139
Metabolic syndrome components:			
Low HDL---cholesterol	45.8%	60.9%	0.219
High triglycerides	55.9%	56.5%	0.961
Abdominal obesity	96.6%	91.3%	0.316
Hypertension	93.2%	73.9%	0.016
Hyperglycaemia and diabetes	69.5%	56.5%	0.614
Sum of metabolic syndrome components	3.61 (0.67)	3.39 (0.72)	0.219
Ever smoking	44.1%	52.2%	0.376
APOE4 carriers	11.9%	17.4%	0.509
Daily energy intake in kcal (mean, SD)	2147.8 (532.07)	2303.06 (395.19)	0.209

^*^Obtained by Student’s T---test, Mann---Whitney or Chi---quared test, as appropriate

Covariance analyses adjusted for Framingham risk score, hypertriglyceridemia, diabetes, BMI, education years, HADS depression score and APOE4 status revealed that physically active participants scored better in mean global and frontal cognitive composites compared to non-active participants. As shown in Table 2, differences in the global and the frontal composite scores were 0.254 (95% CI 0.032 to 0.477, *p* = 0.026) and 0.375 (95% CI 0.110 to 0.639, *p* = 0.006), respectively for physically active vs. non-active participants. No differences were observed in the memory or language cognitive domains. Adjusted results for single tests are presented in Table 3.

**Table 2:** ANCOVA results of cognitive domain scores (Z scores) by group (regular physical activity vs. no/low physical activity)

Cognitive Domains	No or low physical activity *N* = 59	Regular physical activity *N* = 23	Adjusted Difference	*P* value^*^
Global cognition	−0.054 ± 0.53	0·224 ± 0·61	0.254 (0.032 to0.477)	0.026
Frontal function	− 0·075 ± 0·62	0·327 ± 0·70	0.375 (0.110 to 0.639)	0·006
Memory	−0·061 ± 0·78	0·15 ± 0·79	0.192 (−0.174 to 0.558)	0·300
Language	2.884 ± 0·25	2.979 ± 0.25	0.103 (−0.13 to 0.218)	0·080

Data are means ± SD or adjusted means (95% CI)

^*^ANCOVA for group differences adjusted for Framingham risk score, diabetes, high triglycerides, BMI, education years, HADS depression score and APOE4

**Table 3:** ANCOVA results of cognitive test scores by group (regular physical activity vs. no/low physical activity)

Cognitive Test	No or low physical activity *N* = 59	Regular physical activity *N* = 23	Adjusted Difference	*P* value^*^
TMT---A	41.10 ± 12.53	38.35 ± 13.27	−1.504 (−7.800 to 4.791)	0.635
TMT---B	95.53 ± 31.63	77.74 ± 24.88	14.398 (−26.662 to −2.133)	0·022
SEMANTIC FLUENCY	18.47 ± 4.99	20.30 ± 5.53	2.033 (−0.349 to 4.414)	0·093
RAVLT TOTAL	51.25 ± 9.14	52.35 ± 8.14	1.526 (−2.630 to 5.681)	0.467
ROCF COPY	33.30 ± 2.52	33.52 ± 1.93	0.185 (−1.013 to 1.382)	0.759
ROCF RECALL	18.11 ± 5.44	20.68 ± 6.20	1.255 (−1.501 to4.010)	0.367
DIGIT SPAN DIRECT	9.12 ± 2.32	10.09 ± 2.50	0.786 (−0.438 to 2.010)	0.205
DIGIT SPAN REVERSE	5.86 ± 1.97	6.74 ± 1.91	0.971 (0.102 to 1.840)	0.029
VOSP LETTERS	19.51 ± 0.57	19.48 ± 0.67	−0.041 (−0.332 to 0.251)	0.782
VOSP NUMBERS	8.92 ± 1.18	8.91 ± 0.95	−0.100 (− 0.642 to 0.443)	0.716
SDMT	40.02 ± 9.74	46.78 ± 12.90	6.182 (1.224 to 11.139)	0.015
STROOP	33.76 ± 8.60	38.61 ± 8.97	5.303 (1.156 to 9.449)	0.013

Data are means ± SD or adjusted means (95%CI)

^*^ANCOVA for group differences adjusted for Framingham risk score, diabetes, high triglycerides, BMI, education years, HADS depression score and APOE4

## Discussion

The present results suggest that overweight/obese older adults with MetS at high risk for cardiovascular and neurological diseases who regularly engage in aerobic physical activity obtain higher scores in cognitive tests, independently of known cognition-related confounders (age, sex, BMI, educational level, diabetes, depression, APOE genotype) and cardiovascular risk factors. These findings are in line with most previous studies, which show that physical activity is beneficial for cognitive function in older adults [20, 21, 39, 40], although other studies have not found such an effect [23, 24]. Contrary to some previous reports on domain-specific benefits, we have not identified a significant association between physical activity and memory performance in our sample of overweight/obese older adults with MetS. Numerous studies have described attention and executive function as the cognitive domains most frequently affected by MetS in non-demented older adults [11, 41–44]. In this context, the fact that older adults with MetS who engage in regular weekly physical activity of moderate to vigorous intensity obtain better scores in frontal function tests compared to those with no or low physical activity levels is particularly relevant, and it could be argued that physical activity in these subjects helps maintain frontal function or counteracts the adverse effects of MetS on the cognitive areas commonly affected.

The biological mechanisms driving the harmful effects of MetS on cognition are not yet fully understood, but it is known that both cerebrovascular health [45] and structural integrity and connectivity of the brain [46] are compromised in individuals with MetS. Physical activity [47] and exercise [48–51] could exert beneficial effects on cognition in individuals with MetS by simultaneously improving cerebrovascular reserve (i.e., promoting angiogenesis, enhancing the capacity of brain blood vessels, increasing cerebral blood flow, improving oxygen and glucose delivery and modulating inflammation) and brain reserve (i.e., contributing to neurogenesis and synaptogenesis, improving the brain’s structural integrity, volume, plasticity and connectivity). In order to understand how physical activity might positively impact cognition, future studies should aim to unveil the cellular and molecular mechanisms that contribute to the maintenance of brain health focusing on older adults, and assess the interplay between markers of brain health, cognitive health and lifestyle factors (diet, physical activity, sleep quality, living environment, etc.) in aging populations, as this could potentially open the door to new preventative strategies or treatments for cognitive impairment.

At present, the global population is ageing and the incidence of age-related conditions is growing dramatically. As an example, the number of people living with dementia worldwide was estimated at 35.6 million in 2012, and is predicted to double by 2030 and more than triple by 2050 [1]. However this projection might be an overestimation since recent UK population cohort studies have reported a decrease of 1.8% in dementia prevalence [52] and a 20% drop in dementia incidence in the population aged 65 and above over two decades [53], thus suggesting that dementia prevalence will not increase and will remain relatively stable.

As a consequence of the longer lifespan and increasing numbers of older persons, the number of individuals requiring medical attention, social care and support to continue to live well independently will rise, placing additional pressure on national health care budgets and posing a direct threat to social and public health services [54]. In the context of global population ageing and the fact that obesity and MetS are epidemic at present in Western populations, the finding that overweight/obese older adults with MetS who practice regular physical activity obtain overall better cognitive scores compared to non-active individuals suggests that engaging in physical activity could be a useful strategy in preventing or delaying the onset of age-related cognitive impairment and dementia in a high risk population.

Strengths of our study include the use of a comprehensive battery of neuropsychological tests (as opposed to other studies relying solely on a single screening test, such as the MMSE) which allowed us to work with cognitive domain composite scores that are more robust and stable measures of cognitive performance, the focus on an understudied but increasingly prevalent population of older adults with overweight/obesity at high risk for developing chronic age-related conditions, and the inclusion of confounding variables in the analyses (such as education, APOE4 status and depressive symptomatology) that have been overlooked in other studies. We also acknowledge the limitations of our study, including its cross-sectional design, which does not permit to establish a cause-and-effect relationship, the small sample size and insufficient power to detect some effects, the lack of data on inflammatory biomarkers and the use of self-reported data on aerobic physical activity. Having objective physical activity measurements for all participants (e.g. accelerometer data) would have been preferable to avoid any possibility of misclassification bias, but it was not available.

## Conclusion

The present findings support the hypothesis that overweight/obese older subjects with MetS who engage in regular physical activity perform better in a cognitive battery compared to individuals with no or very low physical activity. While our results are the derived from cross-sectional data and therefore do not allow for establishing causality, there is enough evidence in the literature to consider physical activity as a good therapeutic target for age-related cognitive decline risk modification due to its low cost, low risk and accessibility.

## Data Availability

There are restrictions on the availability of data for the PREDIMED-PLUS study, due to the signed consent agreements around data sharing, which only allow access to external researchers for research following the project purposes. Requestors wishing to access the PREDIMED-PLUS trial data used in this study can request to the corresponding author on reasonable request. The request will then be passed to members of the PREDIMED-PLUS Steering Committee for deliberation.
